# Health Inequities among Lesbian, Gay, and Bisexual Adults in North Carolina, 2011–2014

**DOI:** 10.3390/ijerph14080835

**Published:** 2017-07-25

**Authors:** Melissa M. Barnhill, Joseph G. L. Lee, Ann P. Rafferty

**Affiliations:** 1 Department of Public Health, Brody School of Medicine, East Carolina University, Greenville, NC 27834, USA; barnhillm14@ecualumni.ecu.edu (M.M.B.); raffertya@ecu.edu (A.P.R.); 2 Department of Health Education and Promotion, College of Health and Human Performance, East Carolina University, Greenville, NC 27858, USA

**Keywords:** homosexuality, Behavioral Risk Factor Surveillance System, public health surveillance, North Carolina, sexual minority, health status disparities

## Abstract

Inequalities in health have been identified for lesbian, gay, and bisexual (LGB) populations nationally. Policies in the U.S. South offer fewer protections for LGB people than in other regions, yet, limited data exist for this region. North Carolina (NC) BRFSS data from 2011 to 2014 were combined (LGB *n* = 604; heterosexual *n* = 33,170) and analyzed using SAS survey procedures to estimate health characteristics by sexual orientation within gender. Many examined indicators were not different by sexual orientation, however, other results were significant and consistent with findings from state population surveys in other regions of the country. Both genders showed inequities in mental health, having over twice the odds of five or more poor mental health days in the past month and of having ever been diagnosed with a depressive disorder. Sexual minority women had higher odds compared with heterosexual women for ever having smoked cigarettes, current smoking, exposure to secondhand smoke both in the workplace and at home, and both alcohol risk factors, binge and heavy drinking. Being part of the LGB population in NC is associated with worse health. The implementation of anti-LGB policies in the NC warrants ongoing monitoring of LGB health inequities in NC and in other southeastern states for potential effects on the health and well-being of sexual minorities.

## 1. Introduction

Significant health inequities exist for lesbian, gay, and bisexual (LGB) adults compared to heterosexual adults, including in mental health, sexually transmitted infections, substance abuse, and violent victimization [1]. The disproportionate impact of these inequities falls on the approximately quarter of a million North Carolinians who identify as LGB [2]. Inequities in health for LGB people are most commonly posited to exist because of psychosocial stress, discrimination, and stigma unique to this population [3]. More specifically, internalized homophobia, effort required to conceal sexual orientation or gender identity, and family rejection are common proximal sources of psychosocial stress [3]. These sources of stress are supplemented by more distal violence, personally-mediated discrimination, and institutional discrimination or structural stigma [4,5]. Others use a social-ecological approach to note the role of place [6], healthcare, community organizations [7], and the media environment [8,9] in the production of these health inequities.

Substantial changes have led to legal recognitions and increased societal inclusion of LGB people over the last decade. These changes have not been distributed across the United States equally [10]. The U.S. South, and North Carolina (NC) in particular, represent very different policy and social environments than the other states that have previously added sexual orientation questions to their state public health surveillance systems [11,12,13,14]. The South is unique with both a long history of resisting advances in civil rights as well as of LGB organizing for recognition [15]. The same is true for NC [16,17].

A Healthy People 2020 goal is to “improve the health, safety, and well-being of (LGB and transgender) people” [18]. Unfortunately, data on gender identity and transgender status were not available for this study; we thus focus on LGB adults. While one study reported on health inequities among the NC LGB population using 2011 BRFSS data [19], that study was limited by a small sample size of 161 LGB participants. The objective of this research was to combine responses from the 2011–2014 NC Behavioral Risk Factor Surveillance System (NC BRFSS) and estimate gender-specific health inequities by sexual orientation among NC adults.

## 2. Materials and Methods

The Behavioral Risk Factor Surveillance System (BRFSS) is coordinated by the Centers for Disease Control and Prevention (CDC) and comprises annual state-level telephone surveys. The purpose of the BRFSS is to estimate population-based prevalence of adults’ health behaviors, chronic health conditions, and use of preventative services within each state. Interviews are conducted during each month across the year. Respondents are selected using random digit dialing, and since 2011 the sampling frame has included both landlines and cell phones. The annual questionnaire is developed by the CDC, and states can add optional modules or their own questions [20]. North Carolina has participated in the BRFSS since 1987 [21]; four years of data from the NC BRFSS were used for this study. The years 2011–2014 were selected because 2011 was the first year NC included a sexual orientation question in their BRFSS and 2014 was the most recent data that was publicly available at the time of this study.

The primary predictor variable of interest in this analysis was sexual orientation. NC BRFSS included the following state-added question on sexual orientation in 2011–2014, “Do you consider yourself to be heterosexual or straight, homosexual, gay or lesbian, bisexual, or something else?” For the analysis, homosexual, gay or lesbian, and bisexual responses were combined into a dichotomous variable (lesbian, gay, or bisexual (LGB) versus heterosexual or straight). Responses of “something else”, “don’t know”, and “refused” were considered missing and excluded from the analysis.

The health characteristics to be examined were selected based on their inclusion in multiple years of the NC BRFSS during these four years and fell into the domains of health status, chronic health conditions, chronic disease risk factors, health care access and satisfaction, and health care received. If the respondent had refused to answer any question or reported they did not know, the corresponding variable was considered missing and excluded from analysis. A two-level general health status variable was created using responses to the question, “Would you say that in general your health is excellent, very good, good, fair, or poor?” (Poor or fair versus excellent, very good, or good). Five or more days on which physical health was not good was defined by the question, “Now thinking about your physical health, which includes physical illness and injury, for how many days during the past 30 days was your physical health not good?” A similar question was used to define the variable “5 or more days on which mental health was not good”. Ever diagnosed with depression was assessed by asking the respondent if they had ever been told by a doctor, nurse, or health professional that they had a depressive disorder (including depression, major depression, dysthymia, or minor depression). Disability was measured by the NC state-added question, “A disability can be physical, mental, emotional, or communication related. Do you consider yourself to have a disability”?

Chronic conditions (including high blood cholesterol, high blood pressure, diabetes, heart attack, angina or coronary heart disease, stroke, and asthma) were all defined by a positive response to questions asking whether a doctor, nurse, or other health professional had ever told the respondent that they had this condition. A dichotomous cardiovascular disease variable was created by combining responses to the heart attack, angina or coronary heart disease, and stroke questions (ever told they had a heart attack, angina or coronary heart disease, or stroke versus never told they had any of these conditions). Current asthma was determined by asking those ever diagnosed with asthma whether they still had asthma. Body mass index (BMI) was calculated using self-reported height and weight; obesity was defined as a BMI of 30 or greater and overweight or obesity (cumulative overweight) was defined as a BMI of 25 or greater.

Ever smoked cigarettes was defined as having smoked at least 100 cigarettes in their lifetime, and current cigarette smoking as having smoked at least 100 cigarettes and currently smoking every day or some days. Secondhand smoke exposure at home was assessed with a state-added question, “On how many of the past 7 days did someone smoke in your home while you were there?” and exposure to secondhand smoke at work was assessed by a similar question among those who were employed or self-employed. Dichotomous secondhand smoke variables were created (exposed to secondhand smoke on at least one of the past 7 days versus exposed on no days in the past week).

Binge drinking was defined as having five or more drinks per occasion at least once in the past 30 days for men, and four or more drinks per occasion for women. Heavy drinking was defined as consuming on average more than two drinks per day for men and more than one drink per day for women. Fruit and vegetable consumption, respectively, were measured by summing responses to a series of food frequency questions and then dichotomizing based on whether the respondent consumed fruits or vegetables less than once per day in the past month. The frequency and duration of the two physical activities or exercises that the respondent spent the most time doing during the past month were used to estimate minutes of moderate physical activity (or equivalent) done per week; respondents who reported less than 150 min per week were categorized as not meeting aerobic physical activity recommendations. Inadequate sleep was defined as obtaining fewer than 7 h of sleep per day on average by using the question, “On average, how many hours of sleep do you get in a 24-h period”? Inadequate seatbelt use was defined by a dichotomization (always versus nearly always, sometimes, seldom, or never) of responses to the question, “How often do you use seat belts when you drive or ride in a car”?

Having no health insurance was defined by the question, “Do you have any kind of health care coverage, including health insurance, prepaid plans such as HMOs, government plans such as Medicare, or Indian Health Service”? Having no personal doctor was defined as a negative response to “Do you have one person you think of as your personal doctor or health care provider”? Cost preventing medical care was assessed by a positive response to the question, “Was there a time in the past 12 months when you needed to see a doctor but could not because of cost”? Responses to the following question from the CDC optional Health Care Access module were used to define satisfaction with health care received, “In general, how satisfied are you with the health care you received”?

No dental visit in the past year was assessed with, “How long has it been since you last visited a dentist or dental clinic for any reason”? A routine checkup was defined as a general physical exam, not an exam for a specific injury, illness, or condition. Those who reported that their last routine checkup occurred more than 12 months previously were classified as not having had a checkup in the past year. Never having had an HIV test was measured by, “Have you ever been tested for HIV? Do not count tests you may have had as part of a blood donation. Include testing fluid from your mouth”. Similarly, never having had a cholesterol test was measured by, “Blood cholesterol is a fatty substance found in the blood. Have you ever had your blood cholesterol checked”? No influenza vaccination was defined as not having received either a seasonal flu shot or a vaccine sprayed in the nose in the past 12 months. No Pap test in the past three years was defined by two questions, “A Pap test is a test for cancer of the cervix. Have you ever had a Pap test”? and, “How long has it been since you had your last Pap test”?

The demographic characteristics examined included age, gender, race-ethnicity (white, black, Hispanic and other), education, household income, employment status (employed versus not employed), and marital status (married or unmarried couple versus other). The interviewers conducting the NC BRFSS were responsible for determining and reporting gender based on the respondent’s voice, and they only asked the individual if they were unsure.

All questions used in this analysis were asked in each of the four years (2011–2014), except for the following: fruit and vegetable, physical activity, high blood pressure, and high blood cholesterol questions were asked only in 2011 and 2013; pap test and dental visit questions were asked only in 2012 and 2014; sleep and health care satisfaction questions were included only in 2013 and 2014.

The annual response rates for the NC BRFSS ranged from 37.5% in 2012 to 40.5% in 2014 [22,23]. For our analysis, we combined data from the 2011–2014 NC BRFSS and used SAS v.9.4 survey procedures (PROC SURVEYFREQ and PROC SURVEYLOGISTIC) to account for weighting and sample design. All analyses were conducted among men and women separately, and results (except sample sizes) were weighted. We stratified by gender as there are important differences by gender in LGB health [1]. Percent distributions of demographic characteristics were calculated within each of the four gender-sexual orientation groups. For all selected health characteristics, we calculated prevalence rates and 95% confidence intervals among each of the four groups. Adjusted odds ratios (AOR) and 95% confidence intervals (CI) were generated separately for each gender through logistic regressions that adjusted for selected demographic variables based on the statistical significance of their bivariate relationships with sexual orientation. To provide easier comparison with past studies and because of potential estimation errors in conversion [24], we did not convert AORs into prevalence ratios [25]. Because this study used secondary surveillance data, IRB approval was not sought.

## 3. Results

The initial combined year sample size was 35,136, of which 33,170 reported they considered themselves to be heterosexual or straight; 371 homosexual, gay, or lesbian; 233 bisexual; and 96 reported that they considered themselves something else. An additional 306 reported they did not know and 960 refused to answer the question. Responses of “something else”, “don’t know”, and “refused” were eliminated from the analysis, leaving a working sample size of 33,774. Using the two-level sexual orientation analysis variable, we estimated that 2.1% of North Carolina adults identified as LGB and 97.9% were heterosexual.

North Carolina adults of both genders who reported LGB identity were younger (*p* < 0.001) than their heterosexual counterparts, with 33.9% of gay or bisexual (GB) men being aged 18–29 years compared with 20.1% of heterosexual men, and 39.9% of lesbian or bisexual (LB) women versus 16.9% of heterosexual women (Table 1). GB-identified men were better educated than heterosexual men (*p* < 0.001), with only 31.6% of GB men having completed high school or less compared with 46.6% of heterosexuals; 41.3% of GB men had completed some college or technical school while only 29.9% of heterosexual men had completed that level of education. LB-identified women were more likely to be employed than their heterosexual counterparts (55.0% vs. 46.7%, *p* = 0.02). Among both genders, heterosexuals were more likely to be married or part of an unmarried couple compared with LGB people (*p* < 0.001). There were no statistically significant differences among either gender by race-ethnicity or household income.

Results for specific indicators are presented by gender in Table 2 (GB men) and Table 3 (LB women). GB men had higher odds of having five days in the past month on which their physical health was not good (AOR = 1.81; 95% CI = 1.20, 2.73) compared with heterosexual men. LB women had higher odds compared with heterosexual women of reporting fair or poor general health (AOR = 1.68; 95% CI = 1.14, 2.47). Results for both genders showed over twice the odds of five or more days in the past month on which their mental health was not good and of having ever been diagnosed with a depressive disorder. Compared with heterosexual women, LB women had over three times the odds of currently having a disability.

GB men did not have significantly higher odds than heterosexual men for any of the chronic health conditions examined. LB women did have significantly higher odds of having been diagnosed with chronic obstructive lung disease (AOR = 2.15; CI = 1.31, 3.54), ever having been diagnosed with asthma (AOR = 2.23; CI = 1.58, 3.14), currently having asthma (AOR = 2.66; CI = 1.84, 3.86), and having a BMI equal to or greater than 30 (AOR = 1.38; CI = 1.01, 1.89).

The only risk factors examined for which GB men had significantly higher odds than heterosexual men was for secondhand smoke exposure at home (AOR = 1.70; CI = 1.14, 2.55). However, LB women had higher odds compared with heterosexual women for 6 out of the 11 risk factors examined, including all four tobacco smoke risk factors (i.e., ever having smoked cigarettes, current smoking, and exposure to secondhand smoke both in the workplace and at home), and both alcohol risk factors (i.e., binge and heavy drinking).

Among the health care access and satisfaction indicators, GB men had lower odds than heterosexual men for not having a personal doctor (AOR = 0.45; CI = 0.29, 0.70), LB women had lower odds of having no health insurance compared with heterosexual women (AOR = 0.58; CI = 0.38, 0.90). Among screening tests and preventive services, GB men had lower odds of not having had a checkup in the past year, not receiving an HIV test in his lifetime and no influenza vaccine in the past 12 months. LB women also had lower odds than heterosexual women of never having an HIV test (AOR = 0.48; CI = 0.34, 0.68) but they had higher odds of not having gone to the dentist for any reason in the past 12 months.

## 4. Discussion

The findings of this study provide evidence that supports the existing literature that health inequities exist by sexual orientation, which can inform policy and program development in NC. Both LGB men and women had over 2.5 times the odds of ever having been diagnosed with a depressive disorder compared with heterosexuals. These findings are consistent with theories suggesting there is a health cost to having heightened levels of psychosocial stress from discrimination and stigma [3]. Our finding of higher levels of cigarette smoking among LB women was consistent with previous findings [19].

The health inequities found in our study among women are striking. LB women experience higher odds of chronic conditions when compared to heterosexual women. LB women had 3.5 times the odds of having a disability, and over twice the odds of ever having been diagnosed with COPD or asthma, and over 2.5 times the odds of being obese. Stress and obesity [26] have been associated with asthma [26,27], which is consistent with the minority stress model, which posits unique stressors for LGB people [3]. LB women had higher odds of ever smoking cigarettes and current smoking even after adjusting for age, education, and employment status (all of which were significantly related to current smoking in the general population). LB women also had greater odds of exposure to secondhand smoke at work and at home which could be associated with higher rates of asthma. Research that explores a potential association of LB women’s occupational interests [28] and living environments, such as living with a household smoker, is worth noting [29]. Possible explanations of higher rates of obesity among LB women include higher rates of body satisfaction [30] and unwanted sexual contact beginning early in life [31].

We found no significant inequities for GB men regarding smoking, asthma, and alcohol use in NC; however, prevalence of these was consistently higher among GB men. One explanation is that a larger sample size might provide better ability to detect this difference if it exists. Another is that male gender offers some protections for GB men that are less present for LB women. Previous research has noted that bisexual women have some of the highest markers of risk [32,33]. Future research should explore this.

There are significant inequities in indicators of mental health among LGB people in both genders compared with heterosexuals in NC. Previous research has linked these disparities to proximal and distal stressors including structural stigma or institutionalized discrimination [34,35]. It is important to note that NC does not have a statewide law that prohibits LGB discrimination in employment, housing, public accommodations, or credit and lending [10]. Additionally, NC does not have a statewide law that protects individuals from hate crimes based on sexual orientation or gender identity [10]. Health professionals should understand that LGB identity can be a marker of risk for health behaviors and outcomes and be prepared to identify and work against structural causes of these inequities to provide compassionate clinical, gender-specific care to LGB people.

### Strengths and Limitations

Strengths of this study include the combination of four years of data, which provided a larger sample size of sexual minority respondents than the one prior BRFSS-based study of LGB North Carolinians [19], and the use of a high quality public health surveillance data source. Limitations include that BRFSS data are self-reported. The BRFSS may therefore underestimate the proportion of the population who are LGB. There may be misclassification of respondents’ gender in the BRFSS since the assumed gender is recorded by the interviewer and is only asked “when necessary”. Gender misclassification may be related to sexual identity, but it is unclear how it would impact the results. Even with the sample size achieved in this study, we were unable to disaggregate the health characteristics of individuals who identified as lesbian or gay and those who identified as bisexual; this may have attenuated results for bisexual adults [32,33]. Nor could we examine health characteristics by both sexual identity and race/ethnicity. The NC BRFSS did not include questions regarding gender identity, excluding the opportunity for transgender individuals in NC to be identified in the analysis. The NC BRFSS has adopted a gender identity question for 2017.

## 5. Conclusions

There are substantial health inequities for LGB adults living in NC compared to their heterosexual counterparts. Areas of concern include health status and mental health, secondhand smoke exposure, and, for women, respiratory health, obesity, smoking, and alcohol abuse. Yet, in the arena of health care, there were indicators for which LGB adults were more positive than for heterosexuals. To continue making progress, health professionals and public health interventions in NC should consider differences by gender among LGB people. State policymakers could reduce rather than amplify structural stigma and institutionalized discrimination with policy efforts already in place in other areas of the country.

## Figures and Tables

**Table 1:** Demographic characteristics by gender and sexual orientation identity, North Carolina Behavioral Risk Factor Surveillance System, 2011–2014.

Characteristic	Men			Women		
	Gay or Bisexual (*n* = 283) %	Heterosexual (n = 12,847) %	*p*-Value *	Lesbian or Bisexual (*n* = 321) %	Heterosexual (*n* = 20,323) %	*p*-Value *
Total	2.2	97.8		2.1	97.9	
Age, year			<0.001			<0.001
18–29	33.9	20.1		39.9	16.9	
30–44	30.4	26.1		29.3	26.0	
45–59	25.3	27.5		24.0	27.1	
60 or older	10.3	26.4		6.9	30.0	
Race/ethnicity			0.97			0.17
White, non-Hispanic	70.2	68.7		65.7	68.7	
Black, non-Hispanic	18.4	18.6		21.6	20.9	
Hispanic or Latino	7.3	8.3		5.6	6.4	
Other racial group	4.1	4.4		7.1	3.9	
Education			<0.001			0.21
≤high school or GED	31.6	46.6		36.1	40.4	
Some college or technical school	41.3	29.9		33.7	34.8	
College graduate	27.1	23.5		30.1	24.8	
Employment status			0.17			0.02
Employed	55.7	61.2		55.0	46.7	
Not employed	44.3	38.8		45.0	53.3	
Household Income			0.07			0.32
Less than $15,000	16.3	11.0		17.8	14.3	
More than $15,000, less than $35,000	26.1	31.2		32.2	33.9	
More than $35,000, less than $75,000	34.6	29.9		31.7	28.9	
$75,000+	22.9	28.0		18.3	22.9	
Marital status			<0.001			<0.001
Married or part of unmarried couple	25.6	60.6		30.0	55.6	
Not married	74.7	39.4		70.0	44.4	

* *p*-value using Rao-Scott Chi-Square Test.

**Table 2:** Prevalence and 95% confidence intervals (CI) of selected health characteristics among adults by sexual orientation among men, and adjusted odds ratios (AOR), North Carolina Behavioral Risk Factor Surveillance System 2011–2014.

Characteristic	Gay or Bisexual (95% CI) %	Heterosexual (95% CI) %	AOR (95% CI)
Health Status			
Fair to poor general health	15.0 (9.7, 20.4)	19.5 (18.6, 20.3)	0.95 (0.61, 1.47)
≥5 days physical health not good, past 30 days	24.4 (17.7, 31.2)	17.4 (16.5, 18.2)	1.81 (1.20, 2.73)
≥5 days mental health not good, past 30 days	33.1 (25.6, 40.6)	16.3 (15.4, 17.2)	2.23 (1.54, 3.23)
Ever diagnosed with depression	29.8 (22.9, 36.7)	13.3 (12.5, 14.1)	2.60 (1.78, 3.79)
Has a disability	19.7 (13.7, 25.7)	17.2 (16.7, 18.4)	1.28 (0.76, 2.15)
Chronic conditions			
High blood cholesterol (among those tested)	36.4 (24.5, 48.2)	42.4 (40.6, 44.3)	1.09 (0.62, 1.96)
High blood pressure	32.5 (22.3, 42.7)	35.7 (34.1, 37.3)	1.29 (0.78, 2.14)
Diabetes	8.4 (5.5, 11.7)	11.5 (10.9, 12.2)	1.12 (0.70, 1.79)
Heart attack, coronary heart disease, or stroke	6.6 (3.4, 9.7)	11.0 (10.4, 11.7)	0.99 (0.57, 1.71)
Chronic obstructive lung disease (COPD)	7.2 (2.8, 11.6)	6.7 (6.2, 7.2)	1.49 (0.74, 2.97)
Asthma ever diagnosed	12.3 (7.1, 17.4)	9.7 (8.9, 10.5)	1.21 (0.68, 1.85)
Current asthma	6.2 (2.1, 10.5)	5.7 (5.1, 6.3)	1.00 (0.48, 2.07)
Obese (BMI ≥ 30)	25.3 (18.3, 32.4)	29.2 (28.1, 30.3)	0.88 (0.60, 1.29)
Overweight or obese (BMI ≥ 25)	61.1 (53.2, 69.1)	72.0 (70.9, 73.1)	0.71 (0.50, 1.02)
Risk Factors			
Ever smoked cigarettes	47.0 (39.2, 54.8)	53.3 (52.1, 54.5)	0.99 (0.71, 1.38)
Current smoking	29.3 (22.2, 36.4)	22.4 (21.4, 23.4)	1.35 (0.93, 1.95)
Exposed to secondhand smoke at work (among employed for wages), past 7 days	9.1 (3.0, 15.3)	14.1 (12.9, 15.2)	0.66 (0.31, 1.40)
Exposed to secondhand smoke at home, past 7 days	22.6 (15.8, 29.4)	14.6 (13.7, 15.5)	1.70 (1.14, 2.55)
Binge drinking, past 30 days	26.7 (19.4, 34.0)	18.6 (17.6, 19.5)	1.33 (0.90, 1.97)
Heavy drinking, past 30 days	8.6 (4.2, 12.9)	5.7 (5.1, 6.2)	1.44 (0.81, 2.56)
<1 fruit/day	41.6 (26.1, 57.1)	42.2 (39.7, 44.7)	1.04 (0.57, 2.08)
<1 vegetable/day	20.2 (6.6, 33.8)	21.0 (18.9, 23.1)	0.98 (0.41, 2.36)
Did not meet aerobic physical activity recommendations (≥150 min moderate activity/week)	48.0 (36.1, 59.9)	51.5 (49.7, 53.3)	0.91 (0.56, 1.47)
Inadequate sleep (<7 h/day), past 7 days	27.5 (17.3, 37.7)	33.3 (31.7, 34.9)	0.74 (0.44, 1.25)
Does not always wear seatbelt when in a car	13.1 (7.3, 18.9)	11.5 (10.7, 12.2)	1.12 (0.67, 1.89)
Health care access and satisfaction			
No health insurance	23.0 (16.5, 29.6)	19.8 (18.8, 20.9)	1.06 (0.69, 1.64)
No personal doctor	21.5 (14.8, 28.3)	30.2 (29.0, 31.3)	0.45 (0.29, 0.70)
Cost prevented needed care, past 12 months	17.7 (11.6, 23.8)	15.0 (14.2, 15.9)	1.09 (0.69, 1.73)
Not “very satisfied” with health care received	42.2 (30.5, 53.9)	36.0 (34.3, 37.7)	1.21 (0.74, 1.96)
Health care received			
No dental visit, past year	40.9 (30.4, 51.4)	38.4 (36.9, 39.9)	1.23 (0.75, 2.02)
No checkup, past year	24.0 (17.3, 30.7)	31.1 (29.9, 32.2)	0.57 (0.39, 0.84)
No HIV test, lifetime	23.8 (16.7, 30.8)	62.0 (60.8, 63.2)	0.22 (0.14, 0.33)
No cholesterol test, lifetime	14.9 (5.5, 24.4)	20.2 (18.6, 21.8)	0.48 (0.21, 1.10)
No influenza vaccination, past 12 months	53.2 (45.3, 61.0)	59.2 (58.1, 60.3)	0.63 (0.45, 0.89)
No PAP test (women), past 3 years	-	-	-

Note: CI = Confidence Interval, AOR = adjusted odds ratio (adjusted for age, education, employment status). 95% CIs that did not include 1.00 indicated statistical significance at the *p* = 0.05 level.

**Table 3:** Prevalence and 95% confidence intervals (CI) of selected health characteristics among adults by sexual orientation among women, and adjusted odds ratios (AOR), North Carolina Behavioral Risk Factor Surveillance System 2011–2014.

Characteristic	Lesbian or Bisexual (95% CI) %	Heterosexual (95% CI) %	AOR (95% CI)
Health Status			
Fair to poor general health	21.5 (15.6, 27.3)	19.7 (18.9, 20.4)	1.68 (1.14, 2.47)
≥5 days physical health not good, past 30 days	22.0 (16.3, 27.8)	21.5 (20.7, 22.3)	1.34 (0.94, 1.91)
≥5 days mental health not good, past 30 days	40.1 (33.0, 47.3)	22.8 (21.9, 23.6)	2.11 (1.56, 2.84)
Ever diagnosed with depression	42.2 (35.1, 49.3)	22.6 (21.8, 23.4)	2.84 (2.10, 3.84)
Has a disability	28.1 (21.7, 34.5)	16.8 (16.1, 17.5)	3.52 (2.37, 5.22)
Chronic conditions			
High blood cholesterol (among those tested)	25.1 (14.4, 35.7)	40.1 (38.7, 41.6)	0.82 (0.46, 1.46)
High blood pressure	17.9 (9.7, 26.0)	34.8 (33.5, 36.2)	0.87 (0.45, 1.65)
Diabetes	5.5 (2.7, 8.2)	11.1 (10.5, 11.6)	0.87 (0.51, 1.49)
Heart attack, coronary heart disease, or stroke	5.9 (2.7, 9.0)	8.6 (8.2, 9.1)	1.52 (0.83, 2.81)
Chronic obstructive lung disease (COPD)	10.2 (5.9, 14.5)	8.2 (7.7, 8.7)	2.15 (1.31, 3.54)
Asthma ever diagnosed	26.7 (20.2, 33.1)	13.8 (13.1, 14.5)	2.23 (1.58, 3.14)
Current asthma	22.9 (16.7, 29.2)	10.2 (9.6, 11.0)	2.66 (1.84, 3.86)
Obese (BMI ≥ 30)	36.0 (29.1, 42.9)	30.8 (29.9, 31.7)	1.38 (1.01, 1.89)
Overweight or obese (BMI ≥ 25)	61.8 (54.5, 69.1)	61.0 (60.0, 62.0)	1.25 (0.90, 1.72)
Risk Factors			
Ever smoked cigarettes	52.3 (45.2, 59.3)	39.0 (38.0, 39.9)	2.19 (1.64, 2.93)
Current smoking	47.0 (39.2, 54.8)	17.7 (17.0, 18.5)	1.70 (1.21, 2.38)
Exposed to secondhand smoke at work (among employed for wages), past 7 days	8.6 (3.3, 13.8)	4.3 (3.7, 5.0)	2.12 (1.05, 4.26)
Exposed to secondhand smoke at home, past 7 days	26.7 (20.3, 33.1)	13.5 (12.8, 14.2)	2.39 (1.67, 3.41)
Binge drinking, past 30 days	22.6 (16.3, 28.9)	7.9 (7.4, 8.5)	2.35 (1.62, 3.39)
Heavy drinking, past 30 days	8.3 (4.6, 12.1)	4.4 (3.9, 4.8)	1.68 (1.02, 2.77)
<1 fruit/day	37.4 (21.9, 53.0)	39.6 (37.5, 41.8)	0.98 (0.49, 1.95)
<1 vegetable/day	15.1 (3.7, 26.5)	22.4 (20.6, 24.2)	0.64 (0.26, 1.59)
Did not meet aerobic physical activity recommendations (≥150 min moderate activity/week)	48.8 (37.6, 59.9)	54.2 (52.7, 55.7)	0.81 (0.51, 1.29)
Inadequate sleep (<7 h/day), past 7 days	41.4 (30.6, 52.3)	34.0 (32.6, 35.4)	1.31 (0.84, 2.05)
Does not always wear seatbelt when in a car	8.7 (4.9, 12.5)	4.7 (4.3,5.1)	1.58 (0.95, 2.61)
No health insurance	15.3 (10.5, 20.0)	17.5 (16.7, 18.2)	0.58 (0.38, 0.90)
No personal doctor	23.0 (16.7, 29.2)	17.1 (16.3, 17.9)	0.87 (0.59, 1.27)
Cost prevented needed care, past 12 months	27.5 (20.9, 34.2)	20.3 (19.5, 21.1)	1.25 (0.88, 1.76)
Not “very satisfied” with health care received	45.2 (33.8, 56.5)	33.4 (32.0, 34.8)	1.36 (0.85, 2.18)
Health care received			
No dental visit, past year	42.0 (32.5, 51.4)	31.9 (30.6, 33.1)	1.54 (1.02, 2.33)
No checkup, past year	27.4 (21.1, 33.8)	20.8 (20.0, 21.6)	1.13 (0.81, 1.58)
No HIV test, lifetime	30.4 (24.0, 36.8)	57.2 (56.2, 58.2)	0.48 (0.34, 0.68)
No cholesterol test, lifetime	30.5 (19.2, 41.8)	14.0 (12.7, 15.2)	1.44 (0.76, 2.71)
No influenza vaccination, past 12 months	58.0 (50.9, 65.0)	53.1 (52.1, 54.0)	0.90 (0.66, 1.23)
No PAP test (women), past 3 years	22.2 (13.0, 31.3)	20.0 (18.7, 21.3)	1.09 (0.63, 1.88)

Note: CI = Confidence Interval, AOR = adjusted odds ratio (adjusted for age, education, employment status). 95% CIs that did not include 1.00 indicated statistical significance at the *p* = 0.05 level.
